# Abnormal Twinning Behavior Induced by Local Stress in Magnesium

**DOI:** 10.3390/ma15165510

**Published:** 2022-08-11

**Authors:** Dongfeng Shi, Jin Zhang

**Affiliations:** 1 Light Alloy Research Institute, Central South University, Changsha 410083, China; 2 State Key Laboratory of High Performance and Complex Manufacturing, Central South University, Changsha 410083, China

**Keywords:** double twin, local stress state, multiple twin variants

## Abstract

This study investigated the twinning behavior with increasing compressive strain in rolled AZ31 alloy. With that purpose, a polycrystalline structure with an average grain size of 30 μm was utilized to perform the uniaxial compression tests. Microstructure evolution was traced by in situ electron backscattered diffraction (EBSD). Multiple primary twin variants and extension double twins were observed in the same grain. A comprehensive analysis of kernel average misorientation (KAM) and Schmid factor (SF) revealed that the nucleation of twins in one special grain is not only based on the SF criterion, but that it is also strongly influenced by surrounding grains. Moreover, the existing primary twins modified the inner and outer strain distribution close to the twin boundaries. With continued compression, the strain inside the primary twins stimulated the nucleation of double twins, while the strain in the matrixes facilitated twin growth. Therefore, the primary twin growth and the new nucleation of secondary twins could take place simultaneously in the same twinning system to meet the requirements of strain accommodation. Twinning behaviors are controlled by the combined effect of the Schmid factor, strain accommodation between surrounding grains, and variation in the local stress state. The local stress exceeded the critical resolved shear stress (CRSS), implying that twin nucleation is possible. Hence, the twinning process tends to be a response of the local stress rather than the applied stress.

## 1. Introduction

Deformation twinning is recognized as one of the main deformation modes in Mg alloys because it can accommodate strain from the crystal c-axis and improve formability at room temperature by increasing available independent shear systems [1,2,3,4]. As revealed from previous investigations, extension twinning is easy to active and has low critical resolved shear stress (CRSS) among all deformation mechanisms in Mg and its alloys [4,5,6,7]. Moreover, extension twinning will adjust the micro-texture by inducing a crystallographic lattice rotation of 86.3°, which can be activated when the crystal is subjected to tensile stress along the c-axis or compress stress perpendicular to the c-axis in hcp materials. The twinning process can be separated through the following steps: nucleation, propagation, and then growth [8,9,10].

Double twinning is an important twinning behavior to relax strain concentrated on grain boundaries. There are two different double twin structures in Mg alloys: (a) the {10–11}-{10–12} double twin, where the extension twin nucleates inside the contraction twin; and (b) the {10–12}-{10–12} double twin, where the extension twin is generated inside the pre-existing extension twin [11,12]. The {10–11}-{10–12} double twin is regarded as the cause of failure at early deformation stages because of the combination of strain softening and the local generation of twins; it is usually observed when compression strain is conducted on a crystal from the c-axis [13,14,15,16]. In contrast, the {10–12}-{10–12} double twin is perceived to be an enhancer of mechanical properties by adjusting texture and grain refinement, and it is commonly observed when the sample is undergoing a strain path change. Some publications have also provided experimental evidence to confirm the nucleation of {10–12}-{10–12} double twins during complicated metalworking processes at room temperature [10,11,17,18,19]. In addition, other reports have demonstrated that the formation of double tensile twinning is caused by the impingement of two pre-existing extension twins during uniaxial compression tests [8,20]. Most secondary twins were found to be located at the intersections of primary twins. More recently, in single crystals, secondary {101–2} twins with negative SFs were observed, which is attributed to requirements of local strain accommodation.

Twinning nucleation is a complicated activity, and it can easily be affected by many different parameters such as grain shapes, grain sizes, etc. Beyerlein et al. [21,22] proposed that the selection of primary twin variants would be influenced by local stress field induced by neighboring grains in simulations. However, more experimental investigations are needed to strengthen this theory. Moreover, the internal stress caused by the pre-existing twins, in turn, will influence the new nucleation of twin variants with further deformation. However, more investigations need to be conducted to study secondary or ternary twin nucleation. Hence, the local stress state plays an important role in twinning behavior. However, few studies have focused on the nucleation of {10–12}-{10–12} double twins affected by local stress fluctuations.

Therefore, this study assessed the formation of a {10–12}-{10–12} double twin induced by the local stress state at room temperature. To investigate the twinning behavior, an in situ EBSD technique was used to trace the twinning evolution, whereas Schmid factor and KAM analyses were employed to explain it.

## 2. Materials and Methods

In this study, the material employed was a commercial AZ31 (3 wt % Al, l wt % Zn, balance Mg) rolling sheet with a thickness of 20 mm. Compression samples with dimensions of 15 (rolling direction) × 10 (transverse direction) × 10 mm (normal direction) were machined from the rolled sheet. Then, to eliminate dislocations or pre-existing twins, homogenized heat treatment at 250 °C for 24 h was utilized [23,24]. Three comparative mechanical tests were conducted on an AG-X machine (SHIMADZU SPL-10 kN, Kyoto, Japan) at an average strain rate of 10^−3^ s^−1^, and in situ compression tests were interrupted at different deformation stages. Two compression tests were continually conducted along the RD direction. The first sample was compressed along the RD direction at strains of 2% and 4%. The second sample was compressed along the RD at strains of 1.5%, 2.5%, and 3.5%. The other test was performed with two orthogonal paths: rolling and transverse directions. The specimen was first compressed along the RD 1.5%, and then compressed along the TD 2%.

All the samples were prepared by silicon carbide papers ground from 300 to 2000 grit; then, electrochemical polishing with commercial AC_2_ solution (Struers) at a voltage of 20 V and an electric current of 0.1 A for 60 s at a temperature of −20 °C was used to attain mirror surfaces. EBSD examinations were performed using a field emission scanning electron microscope (JOEL JSM 7800F, Akishima, Japan) equipped with an HKL-EBSD system: the step size was 0.6 μm and the magnification was 500. The RD-TD plane was checked via the interrupted EBSD method at the same region several times in three samples. Six possible twinning systems—(−1102) [1–101], (1–102) [−1101], (−1012) [10–11], (10–12) [−1011], (0–112) [01–11], and (01–12) [0–111]—were marked as V_1_–V_6_, respectively [9,12].

## 3. Results

### 3.1. Microstructure and Texture Evolution

**Figure 1:**
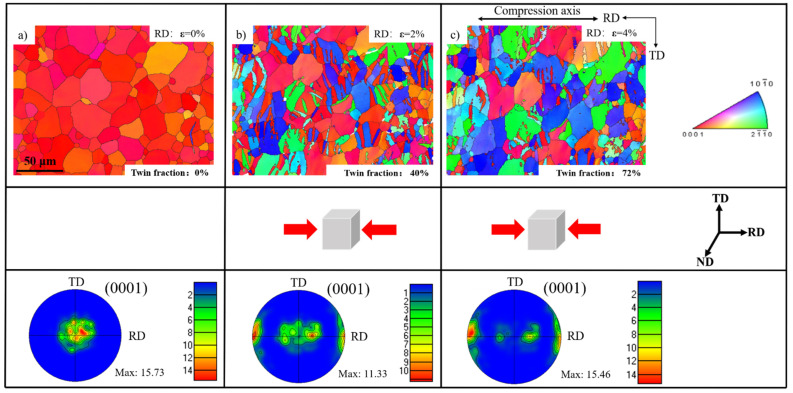
Microstructures and corresponding pole figures at strains of 0% (**a**), 2% (**b**), and 4% (**c**).

In Figure 1, the microstructure and texture evolutions under continued plane compression are presented as in situ EBSD maps and pole figures. The grain boundaries are larger than 15° and presented in black. The IPF map at strain of 0% shows that the average grain size of the polycrystalline is about 30 μm and a typical basal texture with a c-axis parallel to the normal direction shown in the (0001) pole figure. The compression deformation conducted along the RD direction facilitated the nucleation of extension twinning because the c-axis of most grains was perpendicular to the applied stress. Meanwhile, the twin volume fractions were calculated at different strain levels, and the value increased from 0% at strains of 0% to 72% at a strain of 4%. Therefore, the basal texture gradually decreased, and a typical RD texture was generated in the pole figure.

**Figure 2:**
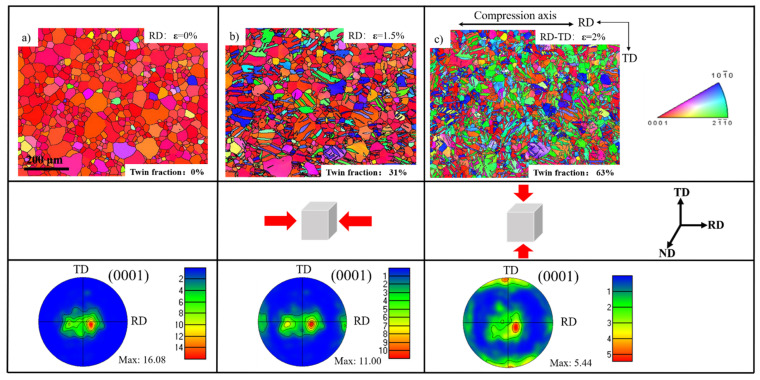
Microstructures and corresponding pole figures at strains of 0% (**a**), 1.5% (**b**), and 2% (**c**).

Different from the uniaxial compression, the strain path change test was conducted on the second specimen. As presented in Figure 2, this sample was first compressed to 1.5% along the RD and then followed by the TD compression to the strain of 2%. First, the RD texture was observed in the pole figure after the first deformation, and then it decreased after the second compression along the TD. Meanwhile, the TD texture was generated. The maximum intensity of the basal texture decreased from 16.08 for the starting material to 11.00 after the first compression along the RD, and it continually decreased to 5.44 after the second deformation along the TD.

**Figure 3:**
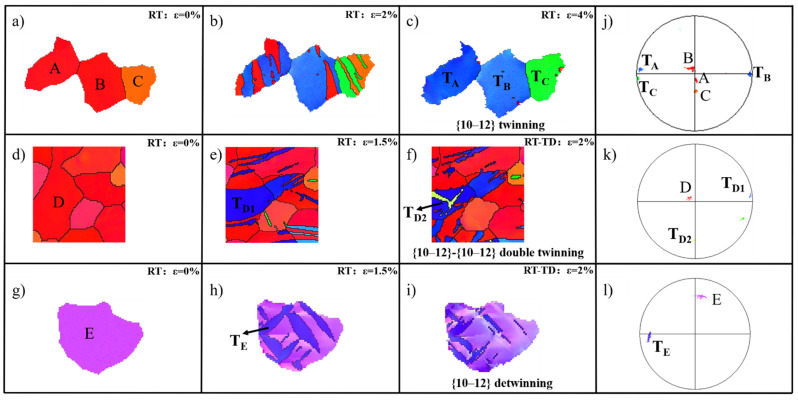
(**a**–**i**) In situ EBSD maps for selected grains at different strain levels; (**j**–**l**) crystallographic orientation of the selected grains and twin lamellae.

As shown in Figure 3, several grains were selected from Figure 1 and Figure 2 and enlarged to analyze the evolution of twinning. Three grains—A, B, and C—were located in the center of the pole figure and identified as the matrix of the starting material. The lamellae T_A_, T_B_, and T_C_ were close to the RD and the misorientation between twin lamellae and corresponding matrixes was close to 86.3°, suggesting that all twin lamellae were {10–12} extension twins. According to the results, twinning behavior is governed by nucleation, propagation, and coalescence mechanisms without changing the loading direction. In Grain D, the primary tensile twin T_D1_ was generated after the first RD compression. When the second strain path changed from the RD to TD, the secondary twin T_D2_ nucleated inside the primary twin T_D1_. Different from the twin morphology in Grain D, no secondary twins were observed in Grain E, although the width of primary twin T_E_ decreased. From the description above, there are two twinning processes in the strain path change test. First, twinning and secondary twinning were the dominate deformation mechanisms in Grain D. Second, twinning and de-twinning governed the deformation in Grain E.

### 3.2. Unusual Secondary Twinning Behavior

**Figure 4:**
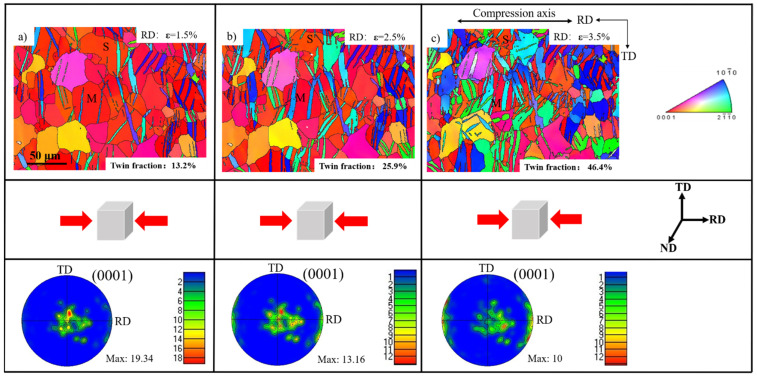
In situ EBSD figures and their corresponding pole figures at different strain levels: (**a**) 1.5%, (**b**) 2.5%, (**c**) 3.5%.

**Figure 5:**
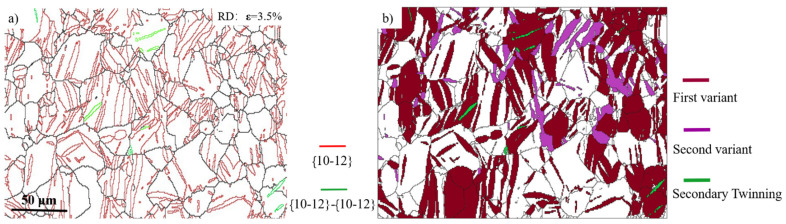
(**a**) The {10–12} primary tension twin and {10–12}-{10–12} secondary twin boundaries are presented in red (86.5° < 11–20 > ± 5°) and green (34° < 11–20 > ± 5°), respectively. (**b**) Primary twins with the first variant and second variant are shown in brown and purple, respectively. In addition, the secondary twins are plotted in green. The two maps correspond to strains of 3.5%.

In some special cases, the twinning growth and secondary twinning nucleation operated simultaneously in the same twinning system during continued RD compression. Figure 4 displays the microstructure and texture evolution at different strain levels. The starting texture with c-axis parallel to the ND decreased and the RD texture was formed at a strain of 1.5% (Figure 4a). As the strain continued to increase to 2.5%, the ND texture experienced further weakening, whereas the RD texture was enhanced and the twin volume fraction is increased from ~13.2% to ~25.9%. After the third compression, the pre-existing twin lamellae grew very fast; several grains were absolutely consumed by twins. The twin volume fraction was enhanced from ~25.9% to ~46.4%. In addition, the texture intensity showed a continued decrease from 19.3 to 10.01. The boundaries of primary tension twins and secondary tension twins are shown in red and green lines in Figure 5a, respectively. The width of twin lamellae expanded very quickly, and a few grains were nearly consumed by the primary twins. Therefore, at early deformation stages, the main deformation mode is the primary twin growth. From Figure 5b, primary twins with two different variants were found in some grains. In addition, the extension twins nucleated inside the existing twins to obtain secondary extension twins under uniaxial compression along the RD direction.

**Figure 6:**
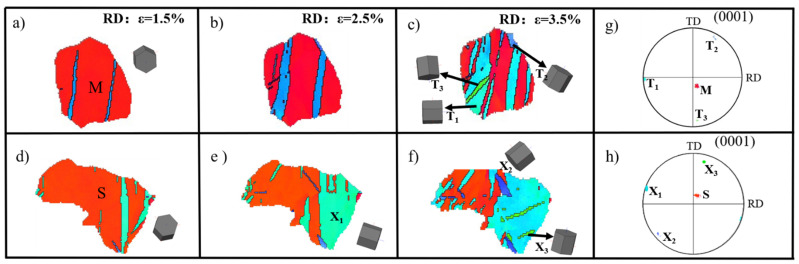
(**a**–**f**) In situ EBSD maps for selected grains at different strain levels; (**g**,**h**) crystallographic orientation of parent grains and twin lamellae.

In order to understand this special twinning behavior, partial maps are enlarged in Figure 6. The microstructural evolution of two selected grains is presented, and the pole figure illustrates the crystallographic relationship between the matrix and twin bands. In Grain M, twins T_1_ and T_2_ are identified as two distinct primary twin variants, V_3_ and V_4_, respectively. The theoretical misorientation angle is about 60°. Similarly, in Grain S, two different twin variants X_1_ and X_2_ are recognized as V_4_ and V_5_, respectively. From in situ IPF maps, the two secondary twins T_3_ and X_3_ nucleated inside the primary tension twin T_1_ and X_1_, respectively. As can be seen from {0001} pole figure, the twins T_2_, T_3_, and X_3_ had a similar crystal orientation and all twins were close to the TD direction, which is different from the direction of the applied stress, indicating that both non-Schmid primary twin variants and secondary extension twins were caused by other parameters. 

## 4. Discussion

Normally, the twinning process is governed by nucleating, growing, and merging mechanisms without changing the loading direction [25,26,27]. The new twins prefer to generate at grain boundaries and at early deformation stage to accommodate the applied stress. With further deformation, a single predominant variant with the highest SF or fewest parallel twins with the same variant grow rapidly until the whole grain is consumed. Sung et al. [28,29,30] reported that the twinning deformation is also affected by changing compressive loadings. In detail, when the compression tests with two orthogonal directions are applied to rolled AZ31 alloy, multiple twinning modes are observed. Therefore, {10–12}-{10–12} double twins can generate inside the primary twin bands. These results are similar to the second sample in this study. Not only were two different kinds of primary variants found in the same grain, but some {10–12}-{10–12} double twins also nucleated inside the primary twins under uniaxial compression. To determine the origin of this behavior, several aspects were considered.

### 4.1. Schmid Factor (SF) Analysis

In Mg alloys, one of reasonable principles for analyzing the formation of twinning is the Schmid factor, which can provide accurate geometrical parameters to judge whether the applied stress meets the requirement of twinning [28,31]. As can be seen from Table 1, both twins T_1_ and X_1_ had the highest SF values among all variants, whereas the twin variants T_2_ and X_2_ ranked with the fifth and third highest SF values (0.10 and 0.26) among all variants, respectively. These twins with SF values lower than 0.3 are identified as non-Schmid twins, indicating that the variant selection is affected by other parameters during compression deformation. Indranil et al. [32,33,34] reported that the stress distributions inside the twin and in front of twin tips are quite different. In addition, the distribution of strain is dependent on the location, i.e., in the center or close to the grain boundary. In Table 1, the primary twin variants are calculated with different assumptive loading directions. The SF values of twin variants in Grain M and Grain S are comparatively low, and some values were negative when compressive loading was performed along the RD direction. This suggests that twins are restricted in this loading condition based on the SF criterion. Compared with two other assumed loading conditions, based on Schmid’s law, it seems more reasonable that twins with high SF values are generated under the assumption of tensile loading along RD. This calculation implies that the secondary twinning behavior is not only governed by the Schmid factor law, but is also influenced by other factors. 

### 4.2. Twinning Behavior Is Affected by Surrounding Grains

**Figure 7:**
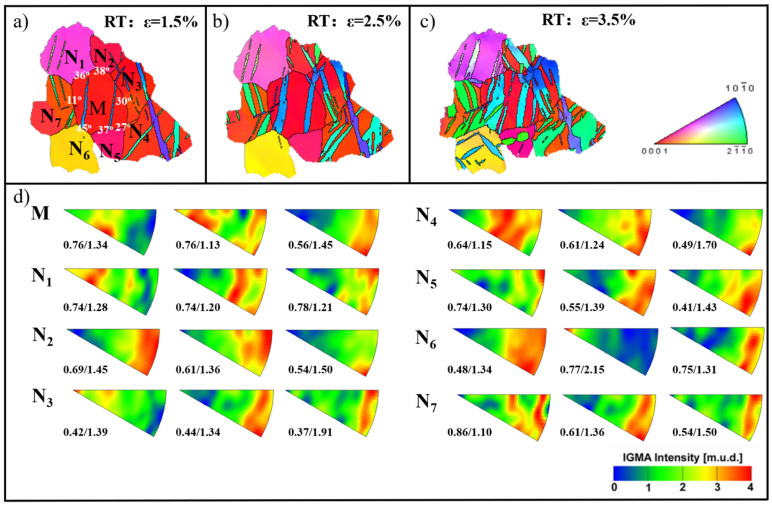
The selected Grain M and seven surrounding grains at different strain levels: (**a**) RD 1.5%; (**b**) RD 2.5%; (**c**) RD 3.5%; (**d**) the maximum/minimum intensities of each IGMA distribution for each grain.

Jonas et al. [17,35] suggested that variant selection is required to accommodate the strain from neighboring grains. Therefore, to understand this special twinning behavior, it was necessary to determine the deformation mechanisms in neighboring grains. Thus, Grain M and seven surrounding grains were selected for study in Figure 7. At a strain level of 1.5%, twins nucleated in all grains except grains N5 and N6. With continued compression, some existing primary twins grew quickly, and the second variants were nucleated as well in some grains. In addition, the double twin nucleated in the center of the primary twin in Grain M and more second variants were generated in surrounding grains at a strain of 3.5%. The misorientation values between Grain M and the neighboring grains are shown in white in Figure 7a, and the average value is 32°. Due to high-angle grain boundaries, the stress is hard to accommodate by twinning and slip transfer. Therefore, the local stress increased in Grain M with increasing compressive strain. In addition, the distributed features of in-grain misorientation axes (IGMAs) in surrounding grains have been investigated [36]. The strong intensities of the IGMAs in Grains N_1_, N_3_, and N_6_ changed from <0001> to <uvt0>, suggesting that the slip mode changed from basal to non-basal. In addition, the strong intensity of the IGMA in Grain M changed, which indicates that the stress inside the grain is variable.

In Table 2, the SF values of twin variants and basal slip in all selected grains are presented. In detail—in the N1, N3, N5, N6, and N7 grains—one or two twin variants with SF values smaller than 0.3 are observed. Generally, the non-Schmid effect is associated with the local stress fluctuations induced by strain accommodation between grains because twinning deformation is a local-stress-controlled nucleation process [17,37]. In addition, the local shear stress of one twinning system and the ability to accommodate the shearing stress from the surrounding grains are two main parameters which affect the nucleation process [1]. The key to achieving new twin variants is that the accumulated local stress reaches the critical resolved shear stress. Similarly, the double twins can nucleate inside the existing primary twins when the accumulated local stress reaches its CRSS. The second variant is considered to be an accommodation mechanism to release the applied stress during uniaxial compression. In this case, the double twins have a similar effect to multiple twin variants.

### 4.3. Local Stress State inside the Grain

Due to the density and spatial configuration of defects, the direction of the stress in the local area was not completely in agreement with the loading stress; it was influenced by grain shapes, sizes, and the segregation of alloy elements and other factors. The local stress state played an important role in deformation mechanisms, especially in twinning behavior.

**Figure 8:**
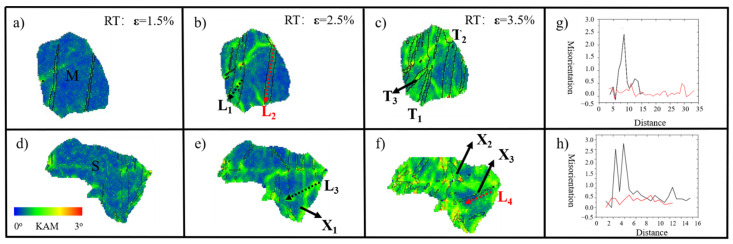
(**a**–**f**) KAM maps with selected grains at different strain levels: (**a**) RD 1.5%; (**b**) RD 2.5%;(**c**) RD 3.5%, (**g**,**h**) point-to-point figures corresponding to L1–L4, respectively.

Previous reports suggested that the macroscopic loading state may significantly deviate from the local stress state when non-Schmid twins are observed [11]. Indranil et al. [33] also reported that geometrically necessary dislocation (GND) densities are in full agreement with KAM values close to grain boundaries. Therefore, the distribution of local stress gradients is in accordance with local misorientation values. The KAM maps at different strain levels and the four L_1_–L_4_ arrows with corresponding point-to-point data profiles are presented in Figure 8. The black and red dotted lines, L_1_ and L_2_, respectively, are selected in two different twin lamellae with the same twin variant as in Grain M. The maximum misorientation in L_1_ is four times larger than that in L_2_, which means that the stress is much larger in twin T_1_ than that in T_2_. The result is that the secondary twin, T_3_, is generated inside twin T_1_ lamellae, although no twins nucleate in T_2_ after further deformation, which seems reasonable. In addition, the lines L_3_ and L_4_ represent point-to-point misorientation values in the same region at strain levels of 2.5% and 3.5%, respectively. The maximum misorientation in L_3_ is six times larger than that in L_4_, which means that the constrained stress is released by the new nucleation of the secondary twin X_3_. It also proves that stress is concentrated on the site where the secondary extension twin is observed. In addition, there are two different primary twin variants in both Grain S and Grain M. From the point-to-point data profile, the distribution of the internal stress is significantly influenced by the growth of two different twin variants. With further deformation, the accumulated flow stress meets the requirement to activate the secondary twins; therefore, the double twins are generated. Usually, several parallel twins will nucleate at the initial deformation stage and the pre-existing twins will grow quickly with further deformation. As mentioned above, from the {0001} pole figure in Figure 6, the twins T_2_, T_3_, and X_3_ have a similar crystal orientation close to the TD direction, and the local stress changes from the RD direction at strain of 2.5% to the TD direction with continued deformation. The existing primary twins change the distribution of strain inside and outside the twin boundaries—i.e., the strain in the primary twins will facilitate the nucleation of double twins—whereas the strain close to primary twin boundaries and inside the matrixes will help with twinning growth. Hence, in Grain M, the primary twin propagation and the double twin nucleation (T_3_) can occur simultaneously in the same twinning system (T_1_).

The selection of twin variants is mainly attributable to the local stress state, which is different with the applied stress and governed by several parameters, such as the strain accommodation between surrounding grains, loading direction, and the shape of grains. Multiple twin variants are generated in one grain when the local stress reaches the value of critical resolved shear stress (CRSS). Similarly, the secondary tension twins can also nucleate inside the primary twins during uniaxial compression. In other words, twinning behavior tends to be a response to accommodate local stress rather than macroscopic stress. Twinning behavior, such as that in double twins and multiple twin variants, requires higher energy and larger applied stress.

## 5. Conclusions

This study investigated the origin of secondary twinning behavior and multiple twin variants which occurred in a rolled AZ31 sheet subjected to uniaxial compression along the RD. An in situ electron backscatter diffraction technique was employed to trace the microstructural evolutions; moreover, SF and KAM analyses were used to study the unusual double-twinning behavior and twin variant selections. The following conclusions can be drawn from this study:

1. Under uniaxial compression, multiple primary twin variants with low SFs are nucleated in one grain because of the local stress fluctuations induced by strain accommodation. Moreover, non-SF twins, in turn, will influence the distribution of local stress gradients.

2. Existing primary twins change the distribution of strain inside and outside the twin boundaries. With further deformation, the strain inside the primary twins will facilitate the nucleation of double twins, whereas the strain close to the primary twin boundaries and inside the matrixes will help with twinning growth. Therefore, twin propagation and the new nucleation of double twins can be activated simultaneously in existing twinning systems.

3. The formation of secondary twins and primary twin variant selections are controlled by a combined effect of the Schmid factor, strain accommodation between surrounding grains, and variation in the local stress state. The key principle to achieve twin nucleation is whether the local stress can reach the value of critical resolved shear stress (CRSS).

These results offer new insights to studies of twinning behavior and can help in the design of new alloys with high strength and ductility properties. Further studies are needed to quantify this double-twinning behavior among all deformation mechanisms.

## Figures and Tables

**Table 1:** Twin variants and their corresponding SF values in the selected grains under different loading directions.

	Compression along RD				Tension along RD		Compression along TD	
SF Values	M	S	T_1_	X_1_	T_1_	X_1_	T_1_	X_1_
V_1_	0.10	0.44	−0.49	−0.39	0.49	0.39	0.25	0.36
V_2_	0.13	0.25	−0.49	−0.44	0.49	0.44	0.03	−0.02
V_3_	0.49	0.02	−0.49	−0.43	0.46	0.43	0.49	0.19
V_4_	0.10	0.45	−0.49	−0.43	0.45	0.43	0.24	0.39
V_5_	0.12	0.26	−0.49	−0.45	0.43	0.45	0.03	−0.01
V_6_	0.47	0.02	−0.49	−0.41	0.46	0.41	0.49	0.16

**Table 2:** Twin variants and their corresponding SFs in the selected grains.

	V_1_	V_2_	V_3_	V_4_	V_5_	V_6_	Active Twin Variant	Basal SF
S	0.44	0.25	0.02	0.45(X_1_)	0.26(X_2_)	0.02	V_4_,V_5_	0.10
M	0.10	0.13	0.49(T_1_)	0.10(T_2_)	0.12	0.47	V_3_,V_4_	0.12
N_1_	0.03	0.08	0.23	0.01	0.10	0.17	V_1_,V_4_	0.45
N_2_	0.40	0.01	0.30	0.38	0.01	0.32	V_1_,V_6_	0.13
N_3_	0.47	0.04	0.24	0.47	0.04	0.24	V_1_,V_3_	0.01
N_4_	0.41	0.01	0.30	0.40	0.01	0.31	V_3_,V_4_	0.10
N_5_	0.11	0.19	0.19	0.11	0.15	0.14	V_1_,V_2_	0.36
N_6_	0.26	0.06	0.04	0.25	0.11	0.10	V_1_,V_4_	0.44
N_7_	0.15	0.10	0.49	0.15	0.10	0.49	V_2_,V_6_	0.01

## Data Availability

The data presented in this study are available on request from the corresponding author.

## References

[1] Barnett M.R., Stanford N., Ghaderi A., Siska F. (2013). Plastic relaxation of the internal stress induced by twinning. Acta Mater..

[2] Balogh L., Niezgoda S.R., Kanjarla A.K., Brown D.W., Clausen B., Liu W., Tomé C.N. (2013). Spatially resolved in situ strain measurements from an interior twinned grain in bulk polycrystalline AZ31 alloy. Acta Mater..

[3] Shi D., Liu T., Zhang L., Hou D., Chen H., Pan F., Lu L. (2016). Localised de-twining in AZ31 Mg alloy sheet during uniaxial compression deformation. Mater. Sci. Eng. A.

[4] Clausen B., Tomé C.N., Brown D.W., Agnew S.R. (2008). Reorientation and stress relaxation due to twinning: Modeling and experimental characterization for Mg. Acta Mater..

[5] Yoo M.H. (1981). Slip, twinning, and fracture in hexagonal close-packed metals. Metall. Trans. A.

[6] Wang F., Sandlöbes S., Diehl M., Sharma L., Roters F., Raabe D. (2014). In situ observation of collective grain-scale mechanics in Mg and Mg-rare earth alloys. Acta Mater..

[7] Boehlert C.J., Chen Z., Gutiérrez-Urrutia I., Llorca J., Pérez-Prado M.T. (2012). In situ analysis of the tensile and tensile-creep deformation mechanisms in rolled AZ31. Acta Mater..

[8] Yu Q., Wang J., Jiang Y., McCabe R.J., Li N., Tomé C.N. (2014). Twin-twin interactions in magnesium. Acta Mater..

[9] Hou D., Liu T., Luo L., Lu L., Chen H., Shi D. (2017). Twinning behaviors of a rolled AZ31 magnesium alloy under multidirectional loading. Mater. Charact..

[10] Qiao H., Guo X.Q., Hong S.G., Wu P.D. (2017). Modeling of {10-12}-{10-12} secondary twinning in pre-compressed Mg alloy AZ31. J. Alloys Compd..

[11] Lou C., Zhang X., Ren Y. (2015). Non-Schmid-based {10-12} twinning behavior in polycrystalline magnesium alloy. Mater. Charact..

[12] Xin R., Guo C., Jonas J.J., Chen G., Liu Q. (2017). Variant selection of {10-12}-{10-12} double twins during the tensile deformation of an AZ31 Mg alloy. Mater. Sci. Eng. A.

[13] Barnett M.R. (2007). Twinning and the ductility of magnesium alloys. Part I: “Tension” twins. Mater. Sci. Eng. A.

[14] Barnett M.R. (2007). Twinning and the ductility of magnesium alloys. Part II. “Contraction” twins. Mater. Sci. Eng. A.

[15] Xu W., Yu J., Jia L., Wu G., Zhang Z. (2021). Deformation behavior of Mg-13Gd-4Y-2Zn-0.5Zr alloy on the basis of LPSO kinking, dynamic recrystallization and twinning during compression-torsion. Mater. Charact..

[16] Wang Y.N., Huang J.C. (2007). The role of twinning and untwinning in yielding behavior in hot-extruded Mg-Al-Zn alloy. Acta Mater..

[17] Jonas J.J., Mu S., Al-Samman T., Gottstein G., Jiang L., Martin E. (2011). The role of strain accommodation during the variant selection of primary twins in magnesium. Acta Mater..

[18] Yildirim D., Tükel S.S., Alptekin Ö., Alagöz D. (2013). Immobilized Aspergillus niger epoxide hydrolases: Cost-effective biocatalysts for the prepation of enantiopure styrene oxide, propylene oxide and epichlorohydrin. J. Mol. Catal. B Enzym..

[19] Shi Z.Z. (2017). Secondary twin variant selection in Mg alloy after a strain-path change. J. Alloys Compd..

[20] Yu Q., Zhang J., Jiang Y. (2011). Direct observation of twinning-detwinning-retwinning on magnesium single crystal subjected to strain-controlled cyclic tension-compression in [0 0 0 1] direction. Philos. Mag. Lett..

[21] Arul Kumar M., Beyerlein I.J., McCabe R.J., Tomé C.N. (2016). Grain neighbour effects on twin transmission in hexagonal close-packed materials. Nat. Commun..

[22] Arul Kumar M., Beyerlein I.J., Tomé C.N. (2016). Effect of local stress fields on twin characteristics in HCP metals. Acta Mater..

[23] Shi D., Liu T., Wang T., Hou D., Zhao S., Hussain S. (2017). {10–12} Twins across twin boundaries traced by in situ EBSD. J. Alloys Compd..

[24] Shi D., Liu T., Hou D., Chen H., Pan F., Chen H. (2016). The effect of twin-twin interaction in Mg-3Al-1Zn alloy during compression. J. Alloys Compd..

[25] Hong S.G., Park S.H., Lee C.S. (2011). Strain path dependence of {1 0–1 2} twinning activity in a polycrystalline magnesium alloy. Scr. Mater..

[26] El Kadiri H., Kapil J., Oppedal A.L., Hector L.G., Agnew S.R., Cherkaoui M., Vogel S.C. (2013). The effect of twin-twin interactions on the nucleation and propagation of {1.0–1.2} twinning in magnesium. Acta Mater..

[27] Khosravani A., Fullwood D.T., Adams B.L., Rampton T.M., Miles M.P., Mishra R.K. (2015). Nucleation and propagation of { 1 0 1 ¯ 2 } twins in AZ31 magnesium alloy. Acta Mater..

[28] Park S.H., Hong S.G., Lee C.S. (2010). Activation mode dependent {1 0–1 2} twinning characteristics in a polycrystalline magnesium alloy. Scr. Mater..

[29] Park S.H., Hong S.G., Lee J.H., Lee C.S. (2012). Multiple twinning modes in rolled Mg-3Al-1Zn alloy and their selection mechanism. Mater. Sci. Eng. A.

[30] Park S.H., Hong S.G., Lee J.H., Huh Y.H. (2015). Texture evolution of rolled Mg-3Al-1Zn alloy undergoing a {10-12} twinning dominant strain path change. J. Alloys Compd..

[31] Hou D., Liu T., Shi M., Wen H., Zhao H. (2018). Deformation Mechanisms in a Rolled Magnesium Alloy Under Tension Along the Rolling Direction. Microsc. Microanal..

[32] Basu I., Ocelík V., De Hosson J.T.M. (2017). Measurement of spatial stress gradients near grain boundaries. Scr. Mater..

[33] Basu I., Fidder H., Ocelík V., Th.M. de Hosson J. (2017). Local Stress States and Microstructural Damage Response Associated with Deformation Twins in Hexagonal Close Packed Metals. Crystals.

[34] Basu I., Ocelík V., De Hosson J.T.M. (2017). Experimental determination and theoretical analysis of local residual stress at grain scale. WIT Trans. Eng. Sci..

[35] Martin É., Capolungo L., Jiang L., Jonas J.J. (2010). Variant selection during secondary twinning in Mg-3%Al. Acta Mater..

[36] Chun Y.B., Battaini M., Davies C.H.J., Hwang S.K. (2010). Distribution characteristics of in-grain misorientation axes in cold-rolled commercially pure titanium and their correlation with active slip modes. Metall. Mater. Trans. A Phys. Metall. Mater. Sci..

[37] Gu X.F., Furuhara T., Kiguchi T., Konno T.J., Chen L., Yang P. (2020). Strain self-accommodation during growth of 14H type long-period stacking ordered (LPSO) structures in Mg-Zn-Gd alloy. Scr. Mater..

